# Inflammatory Markers and Duration of Symptoms Have a Close Connection With Diagnosis and Staging of Acute Appendicitis in Children

**DOI:** 10.3389/fped.2021.583719

**Published:** 2021-06-04

**Authors:** Jiaming Lan, Hai Zhu, Qingshuang Liu, Chunbao Guo

**Affiliations:** ^1^Department of Pediatric General Surgery and Liver Transplantation, Children's Hospital, Chongqing Medical University, Chongqing, China; ^2^Ministry of Education Key Laboratory of Child Development and Disorders, Children's Hospital, Chongqing Medical University, Chongqing, China

**Keywords:** acute appendicitis, white blood cell count, C-reactive protein, neutrophil percentage, duration of symptoms

## Abstract

**Background:** For children with acute appendicitis (AA), a clear diagnosis is a challenge. The purpose of this study is to explore whether inflammatory markers in the blood combined with symptom duration are helpful in the diagnosis of acute appendicitis and in predicting the severity of acute appendicitis.

**Methods:** All the selected patients underwent appendectomy between November 10, 2011 and November 15, 2019, in whom preoperative WBCC, CRP, and NE% had been measured in a short time. All patients were divided into two groups: uncomplicated AA and complicated AA, postoperatively.

**Results:** For our standards, 813 patients were selected, 442 of them had complicated AA. The mean [standard deviation (SD)] age for the uncomplicated AA group was 9.78 ± 2.02 years and for the complicated AA group was 9.69 ± 2.16 years (*P* = 0.55). Elevated WBCC, CRP, and NE% had a higher relatively sensitivity in complicated AA than uncomplicated AA especially when WBCC, CRP, and NE% were at normal levels, which had a sensitivity of 100% in uncomplicated AA, but this only applied to nine patients. CRP values were significantly different in three time groups, whether uncomplicated or complicated AA.

**Conclusion:** The combination of WBCC, CRP, and NE% values is very sensitive for the diagnosis of acute appendicitis, and when we predict complicated AA using the CRP value, we also need to consider the time of symptom onset.

## Introduction

Acute appendicitis (AA) is one of the most common acute abdominal diseases in children (1). However, due to atypical symptoms in children, misdiagnosis often occurs. At the same time, the disease progresses rapidly and more complications occur. Furthermore, the risk of progression to perforation in children is higher than in adults, ranging from 20 to 50% (2). Expeditious treatment is “key,” as this cohort of patients have associated higher morbidity (3).

AA is a rapidly progressing disease that can adversely affect patients if it is not accurately diagnosed and properly treated. And mostly luminal occlusion due to various etiologies and bacterial infections of the appendix are held responsible in the pathogenesis of this disease which is mostly seen in adolescents (4). As well as the secretion stagnates in the appendix cavity, it increases the pressure in the appendix cavity, causes the blood circulation obstacle of the appendix wall, causes the ischemia, necrosis and even perforation of the appendix. According to the intraoperative findings and postoperative pathological diagnosis of the appendix, we can divide AA into several types. Surgery is still the first choice for severe AA (5, 6), but the treatment of uncomplicated AA is controversial (3, 6–10). Laboratory tests such as white blood cell count (WBCC) and C-reactive protein (CRP) have long been used to support clinical data in the decision-making process (1, 5, 11–13). And neutrophil percentage is also used in the diagnosis of AA (14, 15).

The inflammatory response to AA is progressive (16), white blood cell count and C-reactive protein are also thought to change over time (16, 17). This study aimed to analyze the individual and combined values of the white blood cell count (WBCC), C-reactive protein (CRP), neutrophil percentage (NE%) levels, and durations of symptoms in the diagnosis and staging of AA in a large series of children.

## Methods and Patients

Patients undergoing appendectomy in the General Surgery Department at the single institution of Chongqing Children's Hospital were identified. We reviewed medical records of the surgical patients who underwent appendectomy between November 10, 2011 and November 15, 2019. The study was approved by the ethics committee of children's Hospital Affiliated to Chongqing Medical University. The patients were not required to provide written informed consent for this study. And the information we obtain does not relate to any patient's personal data. All patients were selected for the diagnosis of AA, which was obtained through surgical records and pathological diagnosis. If we find that the diagnosis of intraoperative findings or postoperative histological findings are inconsistent, then we choose a more advanced pathological diagnosis as the final diagnosis. Patients were considered eligible for entry into the study upon meeting the following inclusion criteria: age <18 and >7 years, the first diagnosis was AA with concomitant surgical treatment rather than elective surgical treatment for AA, no antibiotics were given before admission, no severe sepsis, no steroids, or immunosuppressive medication administration. Exclusion criteria included patients who have diseases of the blood system and other infectious diseases, the patient's postoperative pathological specimen showing a normal appendix, and admission of more than 12 h without operating. Standard perioperative variables were collected including age, gender, time interval between symptom onset and admission, time interval from admission to operation, preoperative white blood cell count, C-reactive protein, neutrophil percentage, intraoperative, and pathological diagnosis. The more serious diagnoses were chosen. The time when fever, nausea, vomiting, or abdominal pain was reported by the patients was defined as the time of symptom onset. All blood specimens were obtained after admission, and all patients were tested for blood inflammatory markers. From admission to surgery, we used antibiotics once in our institution (18) (ceftizoxime 50 mg/kg and metronidazole 30 mg/kg). The postoperative antibiotic treatment was continued in cases with perforation.

Blood specimen was measured by an automated hematology analyzer (Sysmex, XS-800i). Reference intervals for WBCC were dependent on the age of patients as previously reported (1) (Table 1), and the peak incidence occurs between ages 11 and 12 years (19). So, our age was set to at least 7 years old, and the below reference limit for WBCC was 12.0^*^10^9^/L for all age groups. The reference range at our institution for an abnormal CRP is any value >8 mg/L. According to the Alvarado score (20), the upper reference limit for NE% was 70%.

**Table 1:** Normal reference values for WBCC according to patient age.

**Age**	**Reference WBCC (10^**9**^/L)**
0-3 d	9.0–30.0
4–7 d	5.0–21.0
8–30 d	5.0–19.5
2–12 mo	6.0–17.5
2–4 y	5.0–15.5
5–6 y	5.0–13.5
7–10 y	4.5–12.0
11–18 y	4.5–11.0

In General, operative and pathologic findings were graded G1 for simple or suppurative AA, G2 for gangrenous AA, G3 for perforated AA or a phlegmon, and G4 for a periappendicular abscess (21). In order to analysis, the studied patients were divided into two groups based on the pathologic grades of AA: uncomplicated AA group, those who had G1, complicated AA group, those who had G2, G3, G4.

The SPSS 24.0 (IBM, Armonk, NY, USA) software was used to perform all the statistical analyses. Means [standard deviations (SDs)] were used to describe the numerical variables. Categorical variables were described as frequencies with percentages and were analyzed with Chi-square test or Fisher exact test, as appropriate. Continuous data were expressed as the means ± (standard deviations), maximum and minimum for normally distributed data, which were tested with the Student's *t*-test or one-way ANOVA test, respectively. Results were considered statistically significant if *p* < 0.05.

## Results

A total of 813 children aged 7–18 years were finally studied after inclusion and exclusion criteria. The number of patients, gender, and mean age for each pathology grade group are shown in Table 2. There were 554 males and 259 females, with a mean age of 9.73 ± 2.10 years. Among them, the uncomplicated AA group was 9.78 ± 2.02 years and complicated AA group was 9.69 ± 2.16 years. The majority of the patients with AA fell into the complicated AA group (*n* = 441, 54.2%). In both groups, the vast majority of patients were boys, and there was no difference in male-female age between the uncomplicated and uncomplicated AA groups.

**Table 2:** Demographics of 813 studied patients.

	**G1**	**G2**	**G3**	**G4**	**Total**
**Pathology grade**					
*N*	371	185	228	29	813
M:F	259:112	123:62	151:77	21:8	554:259
Age (yr, mean ± SD)	9.78 ± 2.02	10.08 ± 2.24	9.40 ± 2.07	9.48 ± 2.05	9.73 ± 2.10
	**Uncomplicated** **(*n =* 371)**		**Complicated** **(*n =* 442)**		***P*****-value**
M:F	259:112		295:147		=0.37
Age (yr, mean ± SD)	9.78 ± 2.02		9.69 ± 2.16		=0.55

The estimated sensitivity of elevation of WBCC, elevation of CRP, elevation NE%, as well as the normal levels of the three inflammatory markers (WBCC, CRP, and NE%) in pathologically confirmed AA is shown in Table 3. Clearly, elevated WBCC had a relatively upper sensitivity in both complicated and uncomplicated AA, elevated CRP had a relatively low sensitivity in uncomplicated AA and upper sensitivity in complicated appendicitis, however elevated NE% had a relatively very high sensitivity in both complicated and uncomplicated AA. It is worth mentioning that when WBCC, CRP, and NE% are at normal levels, patients only exist in uncomplicated groups, and there are no patients in complicated groups. And these patients were in three time groups.

**Table 3:** Sensitivity of elevation of WBCC, elevation of CRP, elevation NE%, and meanwhile normal of three inflammatory marker (WBCC, CRP, and NE%) according to surgical and histopathologic findings.

	**Uncomplicated** **(*n =* 371)**	**Complicated** **(*n =* 442)**	**Total** **(*n =* 813)**
Elevated WBC	292 (78.7%)	370 (83.7%)	662 (81.4%)
Elevated CRP	169 (45.6%)	370 (83.7%)	539 (65.9%)
Elevated NE%	353 (95.1%)	437 (98.8%)	790 (97.1%)
Normal WBC And Normal CRP And Normal NE%	9 (2.4%)	0 (0%)	9 (1.1%)

Analysis of type of AA and elapsed time is shown in Table 4. Analysis of pre-hospital time, a controllable factor, showed there was a significant association between starting the admission 24, 48, and 72 h after symptom onset and complicated AA. Not surprisingly, patients with complicated AA had a higher incidence with the time of symptom onset, as compared with those with uncomplicated AA. And analysis of in-hospital time, a controllable factor, we limited it to 12 h and divided it into two groups. It showed no significant association with the severity of AA.

**Table 4:** Analysis of type of appendicitis and elapsed time.

	**Uncomplicated (*n =* 371)**	**Complicated (*n =* 442)**	***P*-value**
**Pre–hospital time (h)**			
0 < Pt ≤ 24	299 (60.8%)	192 (39.1%)	
24 < Pt ≤ 48	61 (28.3%)	154 (71.6%)	<0.01
48 < Pt ≤ 72	11 (10.2%)	96 (89.7%)	<0.01
**In–hospital time (h)**			
<6	230 (45.4%)	276 (54.5%)	
6 ≤	141 (45.9%)	166 (54.0%)	=0.94

According to pre-hospital time, we divided the patients into three groups. Values of WBCC, CRP, and NE% in children undergoing appendectomy for AA according to surgical and pathologic findings are shown in Table 5. Clearly, patients with complicated AA had higher values of WBCC, CRP, and NE% as compared with those with uncomplicated AA in total and it is statistically significant. Concurrently, patients with complicated AA still had higher values of WBCC, CRP, and NE% as compared with those with uncomplicated AA in three groups. However, there was not a significant association between starting the admission 24 h after symptom onset in WBCC values, though other groups were statistically significant. Finally, found that the range between the maximum value and the minimum value was very high in each group.

**Table 5:** Pre-hospital time was divided into three groups.

**Pre-hospital time (h)**	**Uncomplicated**	**Complicated**	
**0 < Pt≤24**	***n =* 299**	***n =* 192**	***P*-value**
WBCC [10^9^ /L, mean ± SD (min, max)]	15.71 ± 4.58(5.09,35.69)	16.54 ± 4.89(3.88,31.67)	=0.06
CRP [mg/L, mean ± SD (min, max)]	14.59 ± 17.69(8,192)	26.57 ± 28.17(8,193)	<0.01
NE% [mean ± SD (min, max)]	0.87 ± 0.07(0.49,0.97)	0.89 ± 0.06(0.46,0.96)	<0.01
**24 < Pt≤48**	***n =*** **61**	***n =*** **145**	
WBCC [10^9^ /L, mean ± SD (min, max)]	14.81 ± 3.60(7.11,24.77)	16.49 ± 4.64(5.18,27.95)	<0.05
CRP [mg/L, mean ± SD (min, max)]	31.70 ± 29.70(8,144)	58.49 ± 43.02(8,256)	<0.01
NE% [mean ± SD (min, max)]	0.83 ± 0.10(0.45,0.95)	0.89 ± 0.05(0.65,0.96)	<0.01
**48 < Pt≤72**	***n =*** **11**	***n =*** **96**	
WBCC [10^9^ /L, mean ± SD (min, max)]	10.92 ± 3.83(5.11,15.43)	16.22 ± 5.06(2.96,34.89)	<0.01
CRP [mg/L, mean ± SD (min, max)]	34.55 ± 70.31(8,245)	74.54 ± 49.38(8,256)	<0.01
NE% [mean ± SD (min, max)]	0.70 ± 0.18(0.43,0.90)	0.89 ± 0.54(0.60,0.97)	<0.02
**Total**	***n =*** **371**	***n =*** **442**	
WBCC [10^9^ /L, mean ± SD (min, max)]	15.41 ± 4.48(3.88,35.69)	16.39 ± 4.48(2.93,34.89)	<0.01
CRP [mg/L, mean ± SD (min, max)]	18.00 ± 24.03(8,245)	48.10 ± 43.59(8,256)	<0.01
NE% [mean ± SD (min, max)]	0.86 ± 0.09(0.43,0.97)	0.89 ± 0.05(0.54,0.97)	<0.01

*WBCC, CRP, and NE% values in children undergoing appendectomy for appendicitis according to surgical and histopathologic findings*.

The relationship between WBCC, CRP, and NE% values in different periods of time in the same type of AA is shown in Table 6. Clearly, values of WBCC will reduce with the time of symptom onset. It is statistically significant in uncomplicated AA, however it is not statistically significant in complicated AA. Values of CRP will increase with the time of symptom onset in both complicated and uncomplicated AA, and it is statistically significant. Finally, values of NE% will reduce with the time of symptom onset in uncomplicated AA, and it is statistically significant. Concurrently, values of NE% are not obviously different between the three time periods.

**Table 6:** To analyze the relationship of WBCC, CRP, and NE% values in different periods of time in the same type of appendicitis.

	**Uncomplicated**	***P*-value**	**Complicated**	***P*-value**
**WBCC (10**^**9**^**/L, mean ± SD)**				
0 < Pt ≤ 24	15.71 ± 4.58		16.54 ± 4.89	
24 < Pt ≤ 48	14.81 ± 3.60		16.49 ± 4.64	
48 < Pt ≤ 72	10.92 ± 3.83	<0.01	16.22 ± 5.06	=0.85
**CRP (mg/L, mean ± SD)**				
0 < Pt ≤ 24	14.59 ± 17.69		26.57 ± 28.17	
24 < Pt ≤ 48	31.70 ± 29.70		58.49 ± 43.02	
48 < Pt ≤ 72	34.55 ± 70.31	<0.01	74.54 ± 49.38	<0.01
**NE%(mean ± SD)**				
0 < Pt ≤ 24	0.87 ± 0.07		0.89 ± 0.06	
24 < Pt ≤ 48	0.83 ± 0.10		0.89 ± 0.05	
48 < Pt ≤ 72	0.70 ± 0.18	<0.01	0.89 ± 0.54	=0.85

## Discussion

AA is one of the most common acute abdominal issues in pediatric surgery (22, 23). Timely and sensitive diagnosis and treatment of acute appendicitis is necessary in order to avoid septic complications that increase with advanced pathology (16). Some studies have shown that inflammatory factors (such as IL-6, IL-8, CD44, etc.) are helpful for diagnosis of AA (24), and WBCC and CRP are certainly the most widely used (1, 25). At the same time, this study increased NE% for analysis.

Mohammed Elniel et al. pointed out 72 h is the time critical point to operate in AA (8). Sometimes it is suggested that interval appendectomy should be selected in our institution when the ultrasound indicates the periappendicular abscess with symptoms for more than 72 h. So, in this study, patients with symptoms for <72 h were selected, and the risk of AA perforation increasing with the time of symptoms in our study was similar to other studies (8, 16, 17, 21, 26–28). Zaid Al-Qurayshi et al. published a study found that appendectomies performed 1 d after admission or on the weekend are associated with disadvantageous outcomes in 265,972 medical records (29). Therefore, the author believes that the long delay after admission will lead to the increase of pathological grade of appendicitis, which will bias the research results. And we will limit the selection of patients to appendectomy within 12 h after admission. Our data indicate that there is no significant increase in the pathological grade of appendicitis after 6 h of admission and 6–12 h of admission. This suggests that a short delay in appendectomy after admission does not increase the risk of perforation in our study, which was similar to Michelle D. Stevenson's study (30). Of course, we still believe that patients who need operations should be treated as soon as possible. Some studies suggested that uncomplicated AA only needs to be treated with antibiotics (3, 6–10). This suggests that antibiotics are effective in the treatment of AA. This will have an impact on the blood test results after admission if antibiotics are used before admission. So, we had to exclude these patients.

In our study, the sensitivity of elevation of WBCC, elevation of CRP, and elevation of NE% in complicated AA was more than in uncomplicated AA. However, we found that elevated CRP had a relatively low sensitivity in uncomplicated AA and upper sensitivity in complicated AA. It seems that CRP is not a useful inflammatory marker for uncomplicated AA, though it does seem useful for detecting complicated AA. Monsalve et al. (19) in a study of 943 patients who underwent appendicectomy, found similar results in children. At the same time, we found that elevation of NE% had a relatively very high sensitivity in both complicated and uncomplicated AA, and we speculate that it should be related to our reference value standard. In particular, we found that the sensitivity to uncomplicated AA is 100% when the WBCC, CRP, and NE% values are in the normal range. It seems that for uncomplicated AA, normal WBCC, CRP, and NE% values can be an effective predictor. However, some articles pointed out that low levels of inflammatory markers have been suggested to sufficiently rule out AA (31, 32), which is a contradiction with our data. Of course, it is important to note that there are only nine patients in this condition. Interestingly, we were going to have 18 cases when the upper reference limit for NE% was 80%. And among them, 17 patients had uncomplicated AA and only one had complicated AA. This seems to mean that combining low levels of WBCC, CRP, and NE% can effectively predict uncomplicated AA. We suggest that we should be cautious about whether to operate on these patients, unless they have very typical symptoms. We are looking forward to more retrospective and even prospective studies to verify this view.

On the other hand, an elevation of at least one inflammatory marker (WBCC, or CRP, or NE%) appeared to be the most sensitive indicator of AA, with 97.6% in uncomplicated AA, 100% in complicated AA, and 98.9% in total. However, the sensitivity of the three combined tests is extremely high, this does not completely rule out AA. This seems to confirm Jasper J. Atema's viewpoint that no level of inflammatory markers could sufficiently exclude the presence of AA in his article (33).

WBCC value, CRP value, and NE% value are higher in complicated AA than in uncomplicated AA, in accordance with most previous reports (1, 6, 8, 15, 19, 34). And our data also support this view. No surprise, the WBCC value, CRP value, and NE% value are higher in complicated AA than in uncomplicated AA when we were divided into three groups according to the time from symptom onset to admission. However, it should be noted that there was no statistical difference between uncomplicated and complicated AA in the 24-h interval between symptom onset and admission. So, we think that CRP value and NE% value are more predictive than WBCC value in the diagnosis of complicated AA. Some previous reports have also suggested that CRP values are more predictive for complicated AA (3, 4). Interestingly, we noticed that the WBCC value decreased with the duration of the symptoms. A previous study of 183 adult patients undergoing appendectomy reported that the WBCC value increased with the duration of symptoms, peaked at 73 h, and then decreased gradually, they considered that it might be related to the establishment of sepsis (16). We also found that the CRP value increased with the duration of symptoms, whether it was uncomplicated AA or complicated AA. It should be related to the physiological characteristics of CRP itself. In general, the blood CRP concentration increases slowly and reaches its peak after 48 h (35). But we noticed that there were statistical differences in CRP values among the three time groups. Even we found that the CRP value in the 24–48 h interval with the uncomplicated AA group was significantly higher than that in the 24-h interval with the complicated AA group. This means that we should also pay attention to the duration of symptoms when we predict complicated AA based on CRP value. There was no statistical difference in NE% value in the three time groups of complicated AA, it seems to mean that NE% value is more advantageous in predicting complicated AA. We are looking forward to more research to verify their relationship.

The present study has certain limitations that need to be considered when interpreting the data. First, the data were collected locally from a single institution, and as a retrospective study, there are some records that are not so accurate, such as whether antibiotics were clearly used before admission, duration of symptoms, and so on. Second, there were only 11 patients in the group of uncomplicated AA with symptoms lasting from 48 to 72 h, which brought some limitations to our analysis. In addition, we chose children between 7 and 18 years old, which brings some limitations to our analysis of children's AA and age factors.

## Conclusions

The WBCC, CRP, and NE% levels together are very useful laboratory tests for diagnosing AA. A combination of normal WBCC, CRP, and NE% values seems to be very useful in predicting uncomplicated AA, and the severity of AA is related to the duration of symptoms, but a small time interval between admission and operation seems to not have a significant impact on the severity of AA. CRP and NE% values are superior to WBCC value in predicting complicated AA. But when we use CRP value to predict complicated AA, we have to consider the duration of symptoms. In this respect, it seems that the NE% value is better than the CRP value.

## Data Availability Statement

The original contributions presented in the study are included in the article/supplementary material, further inquiries can be directed to the corresponding author/s.

## Ethics Statement

The studies involving human participants were reviewed and approved by the ethics committee of Chongqing Medical University. Written informed consent from the participants' legal guardian/next of kin was not required to participate in this study in accordance with the national legislation and the institutional requirements.

## Author Contributions

JL and QL designed and analyzed the data and evaluated the manuscript. JL, HZ, and CG performed the statistical measurements and analyzed the data. CG and JL analyzed the data and wrote the paper. All authors contributed to the article and approved the submitted version.

## Conflict of Interest

The authors declare that the research was conducted in the absence of any commercial or financial relationships that could be construed as a potential conflict of interest.
